# The appropriate division of data in training of computers to predict physicians’ decision on blood transfusions: a reply to Dr. Sander de Bruyne

**DOI:** 10.1186/s12967-021-02929-9

**Published:** 2021-06-27

**Authors:** Yuanyuan Yao, Jenny Cifuentes, Bin Zheng, Min Yan

**Affiliations:** 1 Department of Anesthesiology, The Second Affiliated Hospital, School of Medicine, Zhejiang University, Hangzhou, 310009 China; 2 Program of Electrical Engineering, Universidad De La Salle, Bogotá, Colombia; 3 Department of Surgery, University of Alberta, Edmonton, Canada

Dr. Francesco Marincola

Editor-in-Chief

**Journal of Translational Medicine**

We have read the letter to the editor, written by Dr. Sander de Bruyne about our paper entitled “Computer algorithm can match physicians’ decisions about blood transfusions” [1]. In this study, as mentioned in the letter [2], we used a multilayer perceptron neural network to predict the appropriateness of intra-operative blood transfusion cases. In this preliminary report, the deep learning algorithm yielded a promising accuracy of 96.8% in a dataset of 4946 patients. Expert anesthesiologists classified 3604 cases as appropriate and 1342 as inappropriate in this dataset. This was completed based on the World Health Organization’s (WHO) guidelines.

In his letter, Dr. Bruyne mentioned a well-known adequate practice to prevent the computer algorithm from overfitting and to accurately evaluate machine learning strategies, which is the separation of the sets of training and validation/test. The danger of not dividing the dataset in the training process is that the model may learn an overly specific function that performs well on the training data, but is less effective in generalizing to data outside training. In lieu of this concern, Dr. Bruyne suggested that not splitting data was a problem in this study. Reading through Python scripts, it seemed to Dr. Bruyne that the model was trained and validated on the same data entries. However, importantly, this was not true. As it can be seen in the supplementary material, the files associated with the training and testing have different names. The data were divided and processed before the neural network implementation and consequently saved in different files. In the study published in the Journal of Translational Medicine, no further description about data division was included. This is because the work was focused on providing an exploratory analysis on clinical data, designed for a wider healthcare audience; it also focused on implementing a general, machine learning based classifier to demonstrate how this algorithm could help physicians to make decisions.

We have provided an in-depth analysis on the machine learning strategies, and their implementation details in another paper [3]. In our second paper, the training and validation datasets were split in 70% and 30% of the total data, similar to our first paper [1, 3]. The second paper was focused on a computer science audience; an analysis of the optimal hyper-parameters for different classifiers (Random Forest, Support Vector Machine, MultiLayer Perceptron Neural Network and Decision Tree Classifier) was included [3]. In addition, the training and cross-validation scores were provided and analyzed in the hyper-parameter setting procedure to avoid any overfitting or bias in the evaluation results [3]. We encourage readers to view this latest study if details regarding the deep learning implementation are required.

## Data Availability

Not applicable.
